# Extracting proteins involved in disease progression using temporally connected networks

**DOI:** 10.1186/s12918-018-0600-z

**Published:** 2018-07-25

**Authors:** Rajat Anand, Dipanka Tanu Sarmah, Samrat Chatterjee

**Affiliations:** Drug Discovery Research Centre, Translational Health Science and Technology Institute, NCR Biotech science cluster, 3rd milestone, Faridabad-Gurgaon Expressway, Faridabad, 121001 India

**Keywords:** Temporal connectivity, Gene targets, High-throughput expression data, PPI networks

## Abstract

**Background:**

Metabolic disorders such as obesity and diabetes are diseases which develop gradually over time in an individual and through the perturbations of genes. Systematic experiments tracking disease progression at gene level are usually conducted giving a temporal microarray data. There is a need for developing methods to analyze such complex data and extract important proteins which could be involved in temporal progression of the data and hence progression of the disease.

**Results:**

In the present study, we have considered a temporal microarray data from an experiment conducted to study development of obesity and diabetes in mice. We have used this data along with an available Protein-Protein Interaction network to find a network of interactions between proteins which reproduces the next time point data from previous time point data. We show that the resulting network can be mined to identify critical nodes involved in the temporal progression of perturbations. We further show that published algorithms can be applied on such connected network to mine important proteins and show an overlap between outputs from published and our algorithms. The importance of set of proteins identified was supported by literature as well as was further validated by comparing them with the positive genes dataset from OMIM database which shows significant overlap.

**Conclusions:**

The critical proteins identified from algorithms can be hypothesized to play important role in temporal progression of the data.

**Electronic supplementary material:**

The online version of this article (10.1186/s12918-018-0600-z) contains supplementary material, which is available to authorized users.

## Background

High throughput technologies like Microarray [1, 2] or RNAseq [3] allows to systematically study a disease condition or how organism is responding to different conditions of the experiment [4]. There are many diseases that progress slowly and develop over time. To study such disease progression, it is important to capture high throughput data at different time points of disease development. Several studies have been done capturing the temporal changes in genes expression in the context of such slowly developing diseases [5–8]. Such temporal data gives the expression of genes at each time point of the experiment. Next step is to find the gene/protein targets which are important in the temporal progression of such data. We recently published a similar work [9] tracking disease progression where the biological processes perturbed at each time point were found and connected using a network of connected biological processes The work analyses progression of data by finding processes perturbed temporally and connecting them resulting in paths, giving proteins of processes of the paths as gene/protein targets. A more precise assessment of importance could be firstly quantitatively defining progression and then quantifying the effect of removal of the protein from the network on progression, which was missing in the previous work [9]. Here, we address this limitation by making a novel algorithm giving a more detailed quantitative assessment of the importance of the protein in question. Knowledge of such targets could aid in the understanding the effect of disease condition at gene/protein level. A related work looking at temporal progression have been done which only uses the expression data to infer the interactions among individual genes such that given initial data, subsequent data could be reproduced but failed to determine such interactions [10].

Here, we hypothesized that finding a matrix of interactions between proteins which reproduces the next time point data from the previous time point data could help in for finding targets important in temporal progression of the data. Systematic deletion of proteins and connections from such a matrix and recalculating the effect of perturbation on later time point data could help in finding proteins whose perturbation had maximal effect. Such proteins could be our desired targets.

We therefore, in this study, used a published temporal microarray data from mice liver progressing towards obesity as it is fed with high fat diet to look at proteins perturbed at different time points [11]. Along with this, we used a database of protein-protein interactions already available in the literature [12] . The use of protein-protein interaction networks to aid the analysis of dynamical biological data have been reviewed [13]. This method to overlay gene expression data on the PPI networks has been used in many studies [5, 7, 14, 15]. For example, a study by Liu et al. [14] uses differential PPIs from PPI network in control and disease states to find differential interactions used as diagnostic biomarkers for disease. Another study by He et al. [15] uses information transmitting from the annotated disease genes to differentially expressed genes to decompose the PPI network into modules to be evaluated as biomarkers for diseases.

We hence used such protein-protein interaction network along with temporal expression data to find a matrix of interactions between proteins. This matrix was found such that it reproduces the next time point data from the previous time point data accurately not done in earlier studies. We then used such connectivity matrix to find protein targets deletion of which resulted in maximal effect on later time points through loss of data reproduction. We then ranked proteins based on their effect. We also applied other published algorithms on our connectivity matrix to find protein targets to compare our gene ranking with those obtained from other algorithms. We further validated our gene ranking using a set of positive protein targets from an independent study.

## Results

### Network traversing to obtain fully connected directed time point specific networks

To find the protein targets important in progression of the temporal data, we constructed a matrix of interactions between proteins which reproduces the next time point data from previous time point data. For this, we used a base protein-protein interaction (PPI) network consisting of 9358 proteins with 126,245 interactions from a database of known interactions between proteins (Materials and Methods: String Database). Along with this, we used a published temporal microarray dataset from the mouse liver after feeding with high fat diet for different time durations spread over a period of 140 days, with mouse fed normal diet as controls (Materials and Methods: Microarray Dataset).

**Fig. 1:**
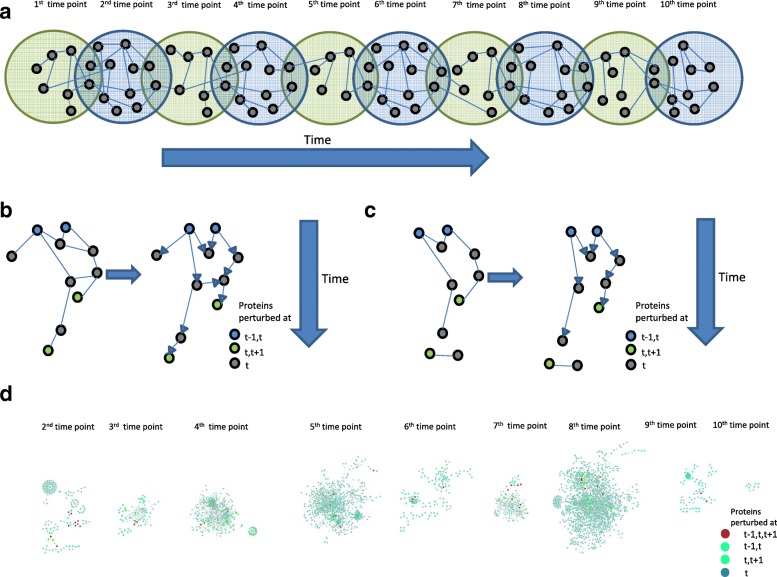
Network Traversing to obtain fully connected directed time point specific networks. **a** Linking time point specific networks using proteins perturbed at consecutive time points. **b** Illustration of giving temporal direction to a time point (say t) specific network, starting from proteins perturbed at t-1,t and reaching proteins perturbed at t,t + 1. **c** Another illustration for a different time point specific network where starting from proteins perturbed at t-1 and t time points, we were not able traverse and reach proteins perturbed at t and t + 1 time points. **d** Resulting fully connected temporally directed time point specific networks

Now, our aim is to prune the interactions of the base PPI network such that given the first time point expression (discretized) data, one can reproduce the next time point data and so on accurately. To find such a matrix of interactions, we hypothesized that the proteins present in consecutive time point specific networks (sub networks from base PPI network such that both proteins of the edges present in the specific network are perturbed in respective time point) could be used as shown in Fig. 1a. That is, starting with proteins perturbed in 1st and 2nd time point, one could traverse the 2nd time point specific network to reach proteins perturbed in 2nd and 3rd time points and again traversing the 3rd time point specific network (Fig. 1a) and so on to reach proteins perturbed at 10th time point and hence making the network. Thus we can obtain a directed network representing the flow of perturbations by connecting the perturbed proteins with their next time point perturbed proteins. Hence, this network can give us an idea of how a perturbed protein at a given time point influences the next time point protein perturbation and hence might predict on a possible mechanistic possibility of perturbations.

Since, traversing a network starting with a set of proteins to reach its first interactors could be represented in matrix multiplication form as (Additional file 1: Text S1. Mathematical representation of traversing a directed network):

1 $$ {b}_{\mathrm{int}}={a}^Tb, $$

with *a* being the adjacency matrix of the network to be traversed, *b* being a vector containing zeros and ones, ones signifying the position of starting proteins and *b*_int_ being a vector containing zeros and nonzeros, nonzeros signifying the position of interactors of starting proteins.

Thus, the whole network traversing as given in Fig. 1a, could be represented as:

2 $$ B\left(t+1\right)={\sum}_{n=0}^{D_{t+1}}{\left({A}_{t+1}^T\right)}^nB(t),t=1:9 $$

Where *B*(*t*)is a vector of size m X 1, with position of non zeros representing that the corresponding proteins are present in network till time *t*, m is the total number of proteins in all the ten networks, *A*_*t*_ is a m X m matrix of zeros and ones with ones representing interaction between proteins pre-sent at t time point taken from the adjacency matrix of network at time t, *D*_*t* + 1_is the diameter of the network at time t + 1.

Now, we aimed to find the matrices *A*_*t* + 1_for each *t* = 1 : 9. For this, first of all, we discretized the expression data by applying a fixed cutoff of 2 log fold change to obtain proteins significantly perturbed at each time point. This choice of cutoff is discussed in Discussion section and also to the fact that we were limited by the number of samples leaving no choice for statistical significance test. Since, the input of the algorithm can be the interaction network and differentially expressed genes, so we chose the 2 log fold change cutoff to start our algorithm. Now, we overlaid the proteins perturbed in 2nd time point on the base PPI network to extract the edges whose both proteins are perturbed in 2nd time point. This gave us the 2nd time point specific network. Similarly, we found all the time point specific networks. The 2nd time point specific network helped us to start with proteins present in 1st and 2nd time points and traverse the network to reach the proteins perturbed in 2nd and 3rd time points to get the traversed proteins as 2nd time point perturbed proteins. We could then use the 2nd time point traversed proteins and traverse the 3rd time point specific network and so on. The adjacency matrices of these time point specific networks are *A*_*t* + 1_ with *t* = 1 : 9.

We observed that some time point specific networks were well connected i.e. starting with proteins perturbed at t-1,t time points, we were able to traverse the t time point specific network to reach the proteins perturbed in t,t + 1 time points (example in Fig. 1b). However, for some networks, we were not able to reach the proteins perturbed in t,t + 1 time points starting with proteins perturbed in t-1,t time points (example in Fig. 1c). Such networks were present in 3rd, 4th, 5th, 7th and 8th time points (not shown). For these networks, we added additional nodes as described below so that in those networks, we can start from proteins perturbed at t-1,t time points and reach proteins perturbed at t,t + 1 time points. For this, we used our base PPI network and a list of total proteins made by taking the union of proteins perturbed at each time point. From this, we constructed a time point independent network by extracting edges from the base network such that both proteins of the chosen edge are present in our list. Then, for the networks at 3rd, 4th, 5th, 7th and 8th time points (say at t time point), we started with proteins perturbed at t-1 and t time points (called source nodes) and traversed the network until there was no further interactor to go to. Then, we took all the traversed proteins of the time point specific network at t time point as source nodes and traversed the time point independent network three times i.e. starting from source nodes, we found its first, second and third interactors. Then, we took these traversed proteins from time point independent network as source nodes and traversed the t time point specific network until there was no further interactor to go to. This exercise gave fully connected time point specific networks i.e. proteins perturbed at t-1 and t time points are connected directly or indirectly to proteins perturbed at t and t + 1 time points. We also gave direction to the edges as we traversed the time point specific networks resulting in 9 fully connected temporally directed time point specific networks shown in Fig. 1d with number of proteins (time point specific perturbed proteins plus additional proteins) and number of edges shown in Table 1. High resolution version is shown as Additional file 2: Figure S2. Some self-interactions could be observed in the figures which were introduced by the algorithm and does not affect our downstream analysis.

### Identifying critical nodes/edges from the directed temporal networks

The above methodology allows us to start with proteins perturbed at 1st and 2nd time point and traverse through the 2nd time point network to reach proteins perturbed at 2nd and 3rd time point, which then are further used to traverse through the 3rd time point network to reach proteins perturbed at 3rd and 4th time point. The procedure is continued until we reach proteins perturbed at 10th time point. We then calculated the fraction of total perturbed proteins that were able to traverse at each time point and found this fraction to be 0.86 averaged across time points (Table 2). This high fraction implies that we are able to accurately represent the temporal data using our PPI networks. Next, we used these time point specific networks to find critical links/nodes involved in progression of our whole data from initial to last time point. For this, we developed an algorithm to find critical links/nodes deleting which we could bring down this fraction of total perturbed proteins traversed. The links/nodes which could block the flow of perturbation from initial to later time point could be important links/nodes in the progression of disease.

#### Identifying critical edges

**Fig. 2:**
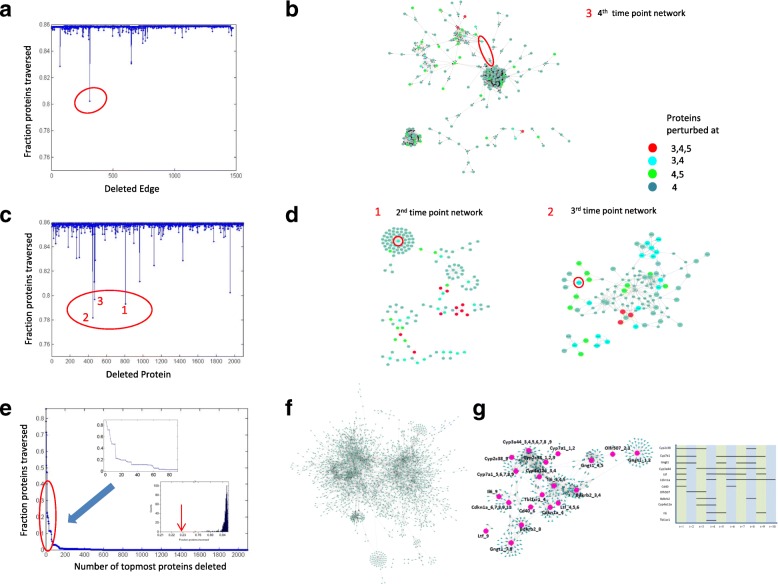
Effect of node and edge deletion on network traversing. **a** Fraction of proteins traversed on deleting each edge with topmost edge (having lowest effect) present in 4th time point network shown in (**a**). (**c**) Fraction of proteins traversed on deleting each node with top 3 nodes (having lowest 2 effect) encircled and the corresponding time point specific network where these are present shown in (**d**) and (**b**). **e** Fraction of proteins traversed on deleting topmost proteins from our ranking with zoomed in plot of deleting top 80 proteins from ranked list shown in inset. Bottom inset shows the distribution of fraction proteins traversed on deleting randomly selected 12 proteins (distribution) and effect when proteins from our ranked list are deleted (red arrow). **f** Single connected network obtained by connecting the 9 time point specific networks. **g** Sub network obtained by overlaying the top 12 proteins on single connected network with the time point where these are perturbed in right panel

We first attempted to find critical links involved in progression. To find such links, we deleted each link one by one and again traversed the whole networks from proteins perturbed at 1st and 2nd time point and checked the number of proteins we are able to traverse and calculated this fraction again. This fraction is plotted in Fig. 2a as a function of the edge deleted. Some edges affect the downstream (later time point) proteins more than others. One such edge is circled in Fig. 2a, is present in the 4th time point network as shown in Fig. 2b. The network is shown without additional nodes for clarity. Network with additional nodes is shown in Additional file 2: Figure S2 third network. A high resolution version of Fig. 2b is uploaded as Additional file 3: Figure S3. The effect of the deleted edge clearly affects its downstream node which has a single incoming edge and further downstream group of proteins which are perturbed in 4th and 5th time points. These affect proteins in network at 5th time point and so on resulting in much large effect compared to deleting other edges. However, the magnitude observed in effect was very less i.e. from initial ~ 0.86 to ~ 0.80. This could be due to the well connectivity of the network and so many proteins will have multiple incoming edges. Thus, deleting a single edge might not be sufficient as the protein downstream of the deleted edge might get traversed through alternative paths. To get a larger effect, we attempted to delete multiple edges simultaneously. Due to large number of edges in each time point specific network, Table 1, even combinatorial searching of edges taking two at a time would be computationally time consuming. So, we attempted to delete each node by one; deleting a node would simultaneously lead to deletion of all its incoming and outgoing edges potentially having a pronounced effect than single edge deletion. This is described below.

**Table 1:** Total number of proteins and edges at each time point specific network

Time Point	2	3	4	5	6	7	8	9	10
Number of Proteins (perturbed+additional)	152	111	443	686	139	160	1196	78	10
Number of perturbed proteins	152	58	230	463	139	72	1105	78	10
Number of edges	188	257	1543	1938	266	399	2585	214	12

**Table 2:** Total perturbed, total traversed proteins and the fraction of traversed to total perturbed proteins at each time point

Time Point	2	3	4	5	6	7	8	9	10
Total Perturbed Proteins	199	58	236	471	174	74	1149	78	36
Total Traversed Proteins	152	58	230	463	139	72	1103	78	10
Fraction	0.76	1	0.97	0.98	0.80	0.97	0.96	1	0.27

**Table 3:** The number of proteins perturbed in t:t + s time points and their nes value of the clustering among top of our ranked list are shown. Nes values with > = 1.3 are italicized

	*t* = 1	*t* = 2	*t* = 3	*t* = 4	*t* = 5	*t* = 6	*t* = 7	*t* = 8	*t* = 9	*t* = 10
s = 0	–	113,0.86	57, 1.06	295, 1.02	531, 0.91	80, 0.98	90,1.05	1102,0.84	32,1.09	6,1.23
s = 1	19,1.14	8,1.22	22,1.14	100,1.04	29,1.01	7,1.28	41,1.12	35,1.07	*2,1.32*	–
s = 2	7,1.24	2,1.22	10,1.23	6,1.23	*3,1.30*	7,1.14	*5,1.30*	–	–	–
s = 3	*2,1.3*	–	*1,1.34*	1,1.28	–	–	*1,1.34*	–	–	–
s = 4	*1,1.37*	–	–	2,1.25	*1,1.43*	*1,1.36*	–	–	–	–
s = 5	–	–	–	–	–	–	–	–	–	–
s = 6	–	–	*1,1.40*	–	–	–	–	–	–	–

**Table 4:** Distribution of total proteins, indispensable proteins and probability of overlap among time points and number of consecutive time points. *p*-values < 0.001 are italicized

	*t* = 1	*t* = 2	*t* = 3	*t* = 4	*t* = 5	*t* = 6	*t* = 7	*t* = 8	*t* = 9	*t* = 10
s = 0	–	113,2,0.0032	57,2,0.1009	*295,10,3.2906E-4*	531,31,0.0075	80,3,0.0653	90,2,0.0150	1102,94,0.0431	32,0,0.0670	6,0,0.6031
s = 1	19,3,0.1322	8,0,0.5104	22,3,0.1637	*100,19,1.8171E-4*	29,6,0.0184	7,0,0.5552	*41,17,3.2091E-9*	35,4,0.1641	2,0,0.8454	0,0,1
s = 2	7,1,0.3412	2,2,0.0065	10,1,0.3791	6,2,0.0694	3,1,0.2044	7,2,0.0894	5,1,0.2882	0,0,1	0,0,1	0,0,1
s = 3	2,1,0.1482	0,0,1	1,0,0.9195	1,0,0.9195	0,0,1	0,0,1	1,1,0.0805	0,0,1	0,0,1	0,0,1
s = 4	1,1,0.0805	0,0,1	0,0,1	2,2,0.0065	1,0,0.9195	1,0,0.9195	0,0,1	0,0,1	0,0,1	0,0,1
s = 5	0,0,1	0,0,1	0,0,1	0,0,1	0,0,1	0,0,1	0,0,1	0,0,1	0,0,1	0,0,1
s = 6	0,0,1	0,0,1	1,0,0.9195	0,0,1	0,0,1	0,0,1	0,0,1	0,0,1	0,0,1	0,0,1

**Table 5:** The number of proteins perturbed in t:t + s time points and their nes value of the clustering among top of the ranking from ‘Collective Influence’ algorithm are shown. Nes values with > = 1.3 are italicized

	*t* = 1	*t* = 2	*t* = 3	*t* = 4	*t* = 5	*t* = 6	*t* = 7	*t* = 8	*t* = 9	*t* = 10
s = 0	–	87,0.92	24,0.51	162,0.95	253,0.87	41,^a^	44,0.91	570,0.91	18,-1.26	2,-0.14
s = 1	12,1.28	2,0.57	9,1.03	67,1.21	13,1.18	*4,1.49*	23,1.25	8,1.22	–	–
s = 2	4,0.92	*2,1.38*	9,1.24	*4,1.38*	*3,1.33*	*3,1.61*	*5,1.40*	–	–	–
s = 3	*2,1.47*	–	*2,1.82*	1,1.25	–	–	*2,1.61*	–	–	–
s = 4	*2,1.30*	–	–	*2,1.48*	*2,1.80*	*2,1.75*	–	–	–	–
s = 5	–	–	–	–	–	–	–	–	–	–
s = 6	–	–	*2,1.77*	–	–	–	–	–	–	–

^a^negative nes values not obtained on randomizations

#### Identifying critical nodes

Next, we attempted to delete each node in the network one by one. The fraction of proteins affected after node deletion is shown in Fig. 2c and suggests three proteins (encircled) whose deletion have a large effect than deleting single edge (See Figs. 2a,c). One of the three protein’s outgoing edge was also significant (showing lowest effect) in our edge deletion analysis (Fig. 2b) as expected. For the other two proteins, the time point specific network where their affect is maximum is shown in Fig. 2d. As can be seen in Fig. 2d left panel, deleting Gngt1 would affect all its downstream proteins (which have a single incoming edge), one of downstream proteins is also present in the 3rd time point, thus potentially affecting 3rd time point proteins also. Similar affect is also seen for the other protein Cyp2c38. A high resolution version for these networks is present in Additional file 2: Figure S2 (two leftmost networks). However, here also, not much decrease in fraction is observed (only from the initial ~ 0.86 to ~ 0.78). This again could be due to the fact that many proteins have multiple incoming edges. Thus, deleting single protein also might not be sufficient as the protein downstream to the deleted protein might get traversed through alternative paths. Here also, to increase the effect, we attempted to delete multiple proteins simultaneously. Due to large number of proteins in each time point specific network, Table 1, combinatorial searching of proteins two at a time would be computationally time consuming.

To mitigate this, we ranked the proteins in increasing order of the fraction (total traversed by total perturbed proteins) obtained on node deletion; topmost protein being one with lowest fraction upon its deletion (Additional file 4: Sheet 1). Then we used this ranking to delete topmost proteins simultaneously from the network and calculate the effect. The number of affected proteins on deletion of topmost proteins simultaneously is shown in Fig. 2e and shows that we are able to attain a large decrease in fraction (from ~ 0.86 to ~ 0.20) by just deleting the top 12 nodes simultaneously (see top inset Fig. 2e). To see the significance of the obtained ranking of proteins, we randomly permuted the ranked list 1000 times and deleted the top 12 nodes each time. A histogram of effects is shown in bottom inset Fig. 2e and the effect from our ranking shown in red arrow. This clearly suggests that proteins chosen randomly, upon deletion, do not give a significantly decreased effect as compared to our originally ranked proteins.

#### Analyzing ranked list of nodes/proteins obtained

To look at how these 12 proteins are together involved in progression, we now extracted the sub networks connecting the 12 proteins. For this we connected the individual time point networks by using the proteins perturbed at consecutive time points (Materials and Methods: Connecting the individual time point networks). This resulted in a single connected network representing all 9 time point specific networks containing 2620 nodes and 7402 edges, Fig. 2f. A high resolution version is shown in Additional file 5: Figure S4. We next overlaid our 12 proteins on this network and found that these proteins were not directly connected with each other (not shown). We checked whether we could connect these 12 proteins by their common interactors and found that through first interactors, we could connect 11 out of these 12 proteins. This implies that these proteins are very closely connected in the network and thus there is a possibility that they have some common functional role. The network is shown in Fig. 2g with 12 proteins along with their time point information in large font for easy readability. The time profile of these 12 proteins is shown in right panel and shows that most of these proteins are perturbed in multiple time points. A high resolution version is shown in Additional file 6: Figure S5.

We also checked whether proteins perturbed in a specific time point are present in the top of our ranked list or not. If specific time point perturbed proteins are present in the top of our list, this could possibly give important time point/s where gene perturbations have a large effect on progression. For this, we checked the position of proteins present in each time point and consecutive time points in our ranked list and quantified the clustering of these proteins towards the top of our list by a nes score [16]. A high nes score would mean that the given set of proteins is statistically significantly concentrated at the top of the ranked list. The distribution of nes values among the time points is shown in Table3 with each element in the table representing total proteins present in t to t + s time points and the nes score of presence of these proteins in top of the ranked list. High nes set of proteins is shown in bold and shows that proteins perturbed at consecutive time points are majorly present at the top of the list. This suggests that proteins perturbed consecutively have a large effect upon their deletion and thus could be important in terms of progression.

Since, our method checks whether removal of a proteins alters the connectivity of network, we wanted to check if there is a degree bias towards topmost proteins or not. For this, since a protein, if perturbed multiple times, might be present multiple times in the network, we found the interactors of each instance of the given protein in the network, took the union of their interactors and used the number of unique interactors to be the total degree of the given protein in the network. The total degree of each protein is given in Additional file 4: Sheet 1. We found that proteins with different degrees varying from low to high degree were present among the top proteins in our ranked list. This suggests the importance of low degree proteins also in giving a large effect upon deletion. These low degree proteins cannot be captured by just using high degree proteins and suggests the importance of our algorithm in capturing such proteins with large effect.

### Applying other network approaches on the temporal network

We further analyzed our single connected network using other existing tools of networks analysis to find targets and checked whether the targets obtained are overlapping with our targets or not. For this, we used two recently published methods for gene target finding: ‘Controllability algorithm’ [17] and ‘collective influence algorithm’ [18]. The ‘controllability algorithm’ takes a directed network as input and finds key dispensable, indispensable and neutral nodes defined as those whose deletion cause a decrease, increase or no change respectively in the minimum number of driver nodes needed to control the PPI network. Driver nodes being those whose control are sufficient to fully control the dynamics of the whole network. Indispensable nodes being more important as these were observed to be overlapping with existing drug targets [19]. Since our network is dynamical, this algorithm, when applied to our network could also identify the proteins involved in progression. The ‘collective influence’ algorithm also takes a single network as input and ranks the proteins in decreasing order of influence such that removing top few proteins disconnects the network. To find such influencers, the algorithm uses percolation theory in random networks to find nodes which minimizes the energy of a many-body system. These top ranked proteins are those which have maximum influence on the network through information flow. The application of this algorithm to our dynamical network could help identify significant proteins involved in maintaining the connectivity of our dynamical network. Hence these proteins could be significant in disease progression.

We first applied the ‘controllability algorithm’ on our directed network to find indispensable nodes, dispensable and neutral nodes. On applying the algorithm, we found that out of total 2620 nodes in our network, 211 (8.05%) were indispensable nodes, 1207 (46.07%) were dispensable and the remaining 1202 (45.88%) were neutral nodes. We next checked the distribution of indispensable nodes among time points. The distribution is shown in Table 4, with each element in the table representing total proteins, indispensable proteins present in t to t + s time points and the hypergeometric probability of the overlap between these proteins. In the table, columns are the time points and rows are the number of consecutive time points perturbed, given by s values. The elements containing *p*-value less than 0.001 and non-zero indispensable nodes are kept in bold. As can be seen, the most significant were proteins perturbed in 4th time point, 4th and 5th time points, and 7th and 8th time points with 10 out of 295 proteins indispensable in 4th time point, 19 out of 100 proteins indispensable in 4th and 5th time points and 17 out of 41 indispensable in 7th and 8th time points. This suggests that these proteins and the corresponding time points where they are perturbed are most important in the network. The indispensable protein names are given in Additional file 4: Sheet 2.

We next applied the ‘Collective Influence’ algorithm on our network. The algorithm gives a ranked list of proteins sorted in decreasing order of their influence such that on removing topmost proteins from network, the networks gets disconnected. We applied the algorithm and chose a value of L = 5 for the analysis and obtained the ranking of proteins. The ranking is given in Additional file 4: Sheet 3. We then sequentially removed the topmost proteins of the ranked list from the network and calculating the size of the giant component and shown in Additional file 7: Figure S6. As can be seen, on removing approximately top 100 proteins the size of the largest connected network becomes half. To check whether there is a statistically significant clustering of proteins perturbed in specific time points in our ranked list, we checked the position of proteins perturbed in specific time points in our ranked list by a nes score. The nes values are shown in Table5 with each element in the table representing total proteins present in t to t + s time points and the nes score of presence of these proteins in top of the ranked list. High nes set of proteins is shown in bold and shows that proteins perturbed at consecutive time points are majorly present at the top of the list.

### Concordance between outputs from three algorithms and validation of ranking

Since the three algorithms were applied on the same single connected network and gives different lists as output, we wanted to find if there is any concordance between the outputs from these three algorithms. Since, the controllability algorithm gave a list of proteins as output and other two: collective influence and combined effect, the algorithm made in this paper, gave ranking of proteins, to check concordance; we checked whether the proteins from controllability algorithm were present in top ranked proteins from two algorithms.

We found an overlap of 37 proteins between 126 proteins from controllability algorithm and top 174 ranked proteins (out of total 1379) from collective influence algorithm which is statistically significant with a *p*-value of 7*10^− 8^ (from hypergeometric distribution). Similarly, we found an overlap of 45 proteins between the 200 proteins from controllability algorithm and top 174 ranked proteins from the algorithm made in this paper which is also statistically significant with a p-value of 2.7*10^− 11^. This suggests that the topmost proteins from both the lists were overlapping significantly with proteins from controllability algorithm.

We finally validated our ranking using external data. Since these proteins have been obtained from a liver microarray data from an experiment tracking obesity over time, we tested the overlap of our list with a positive dataset of obesity disease genes. For this, we used the OMIM database (Hamosh, et al., 2005). From the database we selected genes from ‘obesity’ category resulting in a list of 107 genes and treat them as positive dataset. We found that out of 107 genes, 38 genes were present in our list of 2096 genes. Next, we looked for statistically significant overlap of our topmost genes with positive gene set. For this, we used different topmost gene sets from our list and calculated the significance of overlap of our topmost genes with positive gene set. We found that, for many different topmost genes used up to top 150, we obtained a statistically significant overlap (with p-value of less than 10^-3) of our gene sets with positive gene set, see Additional file 8: Figure S7A. This high statistically significant result thus validates our ranking and shows that our ranked proteins may be involved in the progression of disease thus disease causing.

To see the biological importance of our result, we performed a literature search on out top ranked proteins. Among our top proteins, we found CYPS (Cyp2c38, Cyp7a1, Cyp3a44 and Cyp4a12a) involved which are Cyto-chrome P450 enzymes known to catalyze many reactions involved in synthesis of cholesterol, steroids and other lipids (http://www.genecards.org/cgi-bin/carddisp.pl?gene=CYP1B1) and found that these are mostly perturbed in earlier time points. In addition, we found a protein Cdkn1a perturbed continuously from 6th to 10th time point. The gene encodes a potent cyclin-dependent kinase inhibitor. It binds to and inhibits the activity of cyclin-cyclin-dependent kinase2 or -cyclin-dependent kinase4 complexes, and thus functions as a regulator of cell cycle progression at G1. The protein was shown to be instrumental in the execution of apoptosis following caspase activation. Mice that lack this gene have the ability to regenerate damaged or missing tissue (http://www.genecards.org/cgi-bin/carddisp.pl?gene=CDKN1A). Also mice lacking p21, a protein encoded by Cdkn1a gene were healthy but spontaneous tumours developed and G1 checkpoint control was compromised in cells derived from these mice [20, 21]. We found a protein Foxo1 interacting with both Cyp7a1 and Cdkn1a in the network. Foxo1 is a transcription factor which is a negative regulator of Cyp7a1 transcription [22] and also induces Cdkn1a [23]. The expression of Cyp7a1 was upregulated at 6th time point and expression of Cdkn1a was downregulated at the same time point in our study. This suggests the activation of Foxo1. The activation of Foxo1 usually triggered by insulin [22] and suggests its role in cholesterol metabolism (through upregulation of Cyp7a1) as well as inducing cancerous cells (through downregulation of Cdkn1a) in diet induced obese mice at 6th time point in our study. This validates our top ranked proteins by a match between already known role of these proteins in obesity and the diet induced obesity experimental condition of our data. And this also proivdes a temporal dimension to the mechanisms involved in obesity development.

## Discussion

To study the mechanism of disease development for a disease such as obesity which develops gradually over time, it is important to measure the perturbations of genes of different tissues over time with disease development. However generating such data is not enough and comprehensive analysis of such data is required to capture mechanisms. We aimed here to study a temporal microarray data and develop tools to find critical genes involved in disease development. We developed methods to calculate network of interactions between proteins which reproduces the next time point data from previous time point data. To capture critical proteins involved in disease development from such network, we developed our in-house algorithm, combined effect, and then compared its output with existing algorithms.

Various algorithms are available for extracting critical proteins from a directed or undirected PPI network which does not contain temporal information. However, as discussed above, from measurements of proteins/mRNA at multiple time points, it is important to connect the time point specific networks made with each proteomic/ transcriptomic time point specific data. There is no tool to connect such temporal networks to make a single network that we term as dynamical network. We have attempted here to develop a method to connect time point specific networks made from temporal transcriptomic data as well as giving directions to the edges based on temporal connectivity assumption. This assumption helps making connected directed network amenable to application by algorithms which takes directed network as input. For making the time point specific networks, we used a log fold change of 2 on the expression data to find perturbed proteins mostly because we were getting very dense time point specific networks using log fold change of 1 and prohibited us from getting any block in the flow of perturbations in our perturbation study to rank proteins. Using a log fold change of 2 mitigated this problem and also helped us in working with proteins with high perturbation levels.

The connected directed network allowed us to start with proteins perturbed at 1st and 2nd time point and traverse the network again and again to reach proteins perturbed at last time point and thus calculate the fraction of proteins traversed. We hypothesized that finding nodes/edges deletion of which causes this fraction to become low could block the flow of perturbations from initial to later time point and thus could be important in the progression of disease. To find such nodes/edges, we developed a method that involves removing each node one by one and calculating the fraction of proteins traversed to get a ranking of proteins: top protein being the one whose removal caused maximum decrease in fraction.

We further calculated the properties or our ranking, for example we found that the top ranked proteins are mostly perturbed in multiple time points (Tables 3, 4, 5) and also found both high and low degree proteins towards high ranking proteins. Thus our algorithm captured the low degree proteins also in top ranking proteins which could not be captured by high degree algorithm.

We also applied other published algorithms: ‘Controllability algorithm’ and ‘collective influence algorithm’ on the single connected network. The ‘controllability algorithm’ takes a directed network as input and finds key ‘indispensable nodes’ shown to be overlapping with existing drug targets. The ‘collective influence’ algorithm also takes a single network as input and ranks the proteins in decreasing order of influence such that removing top few proteins disconnects the network. Since both the algorithms take a single network as input, as such these algorithms could not be applied directly on the time point specific networks obtained from the temporal data. Since, our methodology connected the time point specific networks to give a single dynamical connected network, now such algorithms could be applied on our network to find important proteins involved in progression. Other algorithms were applied on our network to check the concordance between gene raking from our algorithm and these algorithms. We found a concordance between the three algorithms outputs: the algorithms gave statistically significantly overlapping lists as output while taking top 174 proteins from ranked lists. We further validated the significance of our ranking using an external database of OMIM genes involved in obesity and showed significant overlap of OMIM genes with genes present in the top of our list.

Whereas the top 174 proteins, when taken gives large overlap between different algorithms tested here, might be large for experimental testing, a much smaller list of proteins (~ 12) from our algorithm could be used initially for experimental testing. These small set of proteins when deleted shows a sharp decrease in fraction proteins traversed as compared to decrease in size of giant component when topmost proteins from collective influence algorithm are removed:12 proteins needed for 60% decrease in effect compared to > 100 proteins required for same decrease in giant component size. We were able to obtain a much smaller list of proteins by the assumption of temporal connectivity: proteins perturbed first are connected to proteins perturbed at later time points in a directed way. This assumption gave a framework to study temporal flow of gene perturbations through the protein networks and gave a quantitative approximation of disease progression. This is in line with ideas from dynamical systems theory where initial gene perturbations give rise to later perturbations through gene/protein interactions [10]. This assumption of temporal connectivity helped us to look at the effect of deleting multiple proteins together on downstream proteins; an affect not seen by deleting single/multiple proteins in an undirected network. Deleting one protein would not allow affecting its downstream protein in PPI network as downstream protein could be reached by other alternative paths in network. However, deleting multiple proteins simultaneously could target multiple paths together and thus observed large effect in our simulations.

We wanted to apply our algorithm to a dense network and hence choose the protein interaction network from the STRING database. The database consists of interactions between proteins from various sources such as coexpression, binding etc. Moreover, the algorithm uses the information of mRNA perturbation of proteins and if there is an association (binding, coexpression etc.) between them, it joins them. Then, it uses such network to mine important edges/nodes. Then, one can go back to STRING database to find what exactly the type of interaction these mined edges represent to design small scale studies. Moreover, the applicability of our algorithm is general in nature and independent of the choice of network.

Our analysis of relevant literature on our top ranked proteins and their temporal profile point to a mechanistic model where initially cholesterol metabolizing enzymes are perturbed and in later time points cell check point control is compromised in mouse level progressing to obesity condition. Thus, apart from already known functions of these proteins, our method, using the dynamical data, correctly prioritized these proteins to shed light on the time points in which these are perturbed (and hence functional) which was not known earlier. To confirm the generality in the application of our algorithm, we analyzed two more temporal datasets from mouse brown adipose tissue and epididymal infiltrating macrophages obtained from GEO database GSE63168 and GSE63171 generated from diet induced obese mice similar to disease conditions of liver tissue analyzed in our study. The resulting ranked gene list is given in Additional file 4 and shows statistically significant overlap with obesity related genes from OMIM database and have biological function related to obesity condition of our experiment (Additional file 1: Text S1. Validation of ranked lists from other temporal datasets). This shows the ability of our algorithm to analyze other gene expression datasets to give a ranking of genes based on importance. Our algorithm can be applied to any temporal high-throughput datasets to find important proteins mediating progression.

## Conclusion

Our methodology takes a temporal transcriptomic response of mouse liver to high fat diet and a PPI network as input to give a raking of proteins. The top ranked proteins from our analysis could be important mediators driving the temporal response of liver to high fat diet. These proteins could be checked in knock out mouse model in high fat diet conditions. Our methodology is generic and could be applied to any temporal transcriptomic/proteomic response from invitro/invivo system.

## Methods

### String database

The base protein-protein interaction network was downloaded from string database version 9.1 taking links with a score greater than 7000 [12].

### Microarray dataset

The microarray data was obtained from an experiment where one group of mice were fed with high fat high sucrose diet (treated group) and another group with normal diet (control group). Both groups of mice were fed respective diets for following days: Day1, Day 6, Day 10, Day 14, Week 0, Week 3, Week 6, Week 9, Week 12, Week 15 and Week 18 before taking tissue samples from both groups of mice. This experiment was repeated for three times. Further details of the experiment are given in [11]. Then, microarray experiment was performed on tissue samples and after suitable normalization of the signal intensities of each probe using Agilent Genespring GX software, three values of log fold change for control sample and treated sample were obtained for each probe and at each time for each tissue. The data used in this study was from liver tissue and downloaded from the NCBI repository under GEO accession number GSE63175 and suitable processing was done. Briefly, out of total ~ 29,411 genes measured, data was processed to filter out genes not perturbed 2 fold even in one time point resulting in a matrix of 19,303 genes across 10 time points. Further details of the processing are given in [9].

### Connecting the individual time point networks

To connect the individual time point networks, we first tagged the proteins uniquely present in different time point specific networks by the time point in which they are perturbed. For proteins present in consecutive time points, the tag contains information about all the respective time points. For example if a protein ‘X’ is perturbed at ‘t’ time point, then it is tagged as ‘X_t’ and ‘Y’ is perturbed at time points t-1,t and t + 1, then the tag contains ‘Y_t-1,t,t + 1’. Following this, we stacked the edge lists of each time point specific network. This way, since the proteins perturbed at consecutive time points have the same tag in time point specific networks, the time point specific networks get connected through the proteins perturbed at consecutive time points in the final edge list. This gives a final tagged network containing 2620 nodes and 7402 edges. The final network is shown in Fig. 2f. A high resolution version is shown in Additional file 5: Figure S4.

## Additional files

Additional file 1: **Text S1.** Mathematical representation of traversing a directed network. Validation of ranked lists from other temporal datasets. (ZIP 302 kb)

Additional file 2: **Figure S2.** High Resolution version of Fig. 1d. (PDF 644 kb)

Additional file 3: **Figure S3.** High Resolution version of Fig. 2b. (PDF 108 kb)

Additional file 4: Ranking of proteins using algorithm made in the paper and collective influence algorithm and results from controllability algorithm. (XLSX 140 kb)

Additional file 5: **Figure S4.** High Resolution version of Fig. 2f. (PDF 817 kb)

Additional file 6: Figure S5 High Resolution version of Fig. 2g left panel. (PDF 172 kb)

Additional file 7: **Figure S6.** Effect of removal of influencing proteins on size of giant component. Size of the giant component of single connected network on removal of topmost proteins from the ranked list sorted in decreasing order based on their influence obtained by applying ‘Collective Influence’ algorithm on single connected network. (PDF 2365 kb)

Additional file 8: **Figure S7.** Significance of overlap of topmost genes with positive gene set. The *p*-value of significance of overlap between topmost genes and positive gene set (fraction of times overlap from random permuted ranked list is greater than actual observed overlap) is plotted against topmost proteins used. (PDF 118 kb)

## Abbreviation

RNA: Ribonucleic acid
