# Supplementary material for: Extracting proteins involved in disease progression using temporally connected networks
Source: BMC Syst Biol. 2018 Jul 25;12:78. doi: 10.1186/s12918-018-0600-z (PMC6060549; doi:10.1186/s12918-018-0600-z)
Supplement: Supplementary file 1 — Text S1. Mathematical representation of traversing a directed network. Validation of ranked lists from other temporal datasets. (ZIP 302 kb) [file 12918_2018_600_MOESM1_ESM.zip › TextS1_Supporting information.docx]

Additional file

**S1 Text. Mathematical representation of traversing a directed network.**

Here, we develop a mathematical representation of network traversing as done in the study, see Eqns 1-2. Briefly, in matrix traversal, we start with a set of proteins on a directed network and traverse the network along their outgoing edges to reach their interaactors.

As an example, we traverse the network shown in Fig.S1A by starting with proteins A,B,C and D, traversing along their outgoing edges to reach their interactors E,F and G. Considering the adjacency matrix ‘a’ of the network as shown in Fig.S1 and a vector ‘b’ containing zeros and ones: ones signifying the position of starting proteins, one can do the matrix multiplication a^T^b to get a vector b_int_ where the position of nonzeros signify proteins E,F and G. That is, matrix multiplication a^T^b gives the vector representing the proteins obtained by traversing the network (with adjacency matrix ‘a’) starting from proteins represented in vector ‘b’ as given in Eqn 1.

Now, consider a directed network with adjacency matrix A and B being the vector with position of ones signifying the position of starting proteins. If the matrix is to be traversed multiple times, i.e. traverse to interactors of starting proteins (along outgoing edges) and again traverse the interactors of resulting interactors and so on, one can do matrix multiplication A^T^B multiple times. For example, for traversal two times, the equation becomes B2=A^T^(A^T^)B. For traversing the matrix multiple times till one reaches the proteins with no further outgoing interactor, we can multiply A^T^ with B multiple times to get BD= A^T^(A^T^(A^T^…)B, with A^T^ being multiplied D number of times: D being diameter of network A. To get total proteins traversed, one can take union of proteins traversed, mathematically represented by taking the sum Bf= B+A^T^B+ A^T^(A^T^)B+ ….A^T^(A^T^(A^T^…)B..D times.

Now, as given in the main text, starting with proteins perturbed at 1 and 2 time points, one can traverse the network at 2^nd^ time point multiple times and take the union of proteins traversed, to give proteins perturbed in 2^nd^ time point. Thus, in above context, as shown in Fig.S1B, with A=A_2_, B=b0, D=D_2_, Bf=proteins present in 2^nd^ time point, we get $B_{f}=b_{0}+\sum_{n=1}^{D_{2}} {{(A}_{2}^{T})}^{n}b_{0}$. With B(t)=proteins present till t time point, one can see that $B_{f}=b_{0}+\sum_{n=1}^{D_{2}} {{(A}_{2}^{T})}^{n}B(1)$. With b_0_=B(1), we can get B_f_=B(2)=$\sum_{n=0}^{D_{2}} {{(A}_{2}^{T})}^{n}B\left( 1 \right)$. For a general t, the resulting equation becomes as shown in eqn(2) in main text.

**S1 Text. Validation of ranked lists from other temporal datasets.**

To vaidate the ranked list of genes from brown adipose temporal dataset, we checked the overlap of total 1766 ranked genes with 107 genes from OMIM database realated to obesity disease. With an overlap of total 34 genes, we found that for many different topmost genes used upto 110, we obtained a statistically significant overlap (p-value of less than 10^-1.3=0.05) Fig.S7B.

We also checked overlap of OMIM disease genes with ranked list of genes from epididymal infiltrating macrophages temporal dataset. Here also we found, for an overall overlap of 33 genes between our 1548 ranked gene list and OMIM genes, we found for many differnet topmost genes upto 127 genes, we obtained a statistically significant overlap (p-value of less than 10^-3) Fig.S7C. This high statistically significant result thus validates our ranking.

To further see the biological importance of our result, we performed a literature survey on the topmost genes from two lists: FileS1: Sheet4,5.

For the ranked list from brow adipose dataset (File S1: Sheet4), we made a literature survey of one of the topmost protein: ADAM17. It has been reported in literature that ADAM17 pathway is associated in the control of glucose homeostasis and hepatic, adipose and vascular inflammation in both nutritional and genetic and models of obesity in mice [1,2], as well as in patients with obesity-related T2DM [3,45]. In addition , heterozygous Adam17+/2 mice are protected from diet-induced obesity and inflammation [6] and Adam17-deficient mice are seen to have hypermetabolic phenotype[7], thus showing its role in obesity condition in brown adipose tissue.

From the ranked list from epididymal infiltrating macrophages dataset (File S1: Sheet5), we made a lietrature survey of one of the topmost protein: ARHGEF11(PDZ-rhogef). This protein plays a fundamental role in numerous cellular processes that are initiated by extracellular stimuli that work through G protein coupled receptors. It has been reported that PDZ-rhogef knockout mice are resistant to diet-induced obesity and insulin resistance[8] thus confirming its role in obesity condition. Our prediction of its perturtbations in epididymal infiltrating macrophages suggests that this protein might be playing role in obesity condition through maccrophages.

**References**

Menghini, Rossella, et al. "Tissue inhibitor of metalloproteinase 3 deficiency causes hepatic steatosis and adipose tissue inflammation in mice." *Gastroenterology* 136.2 (2009): 663-672.

Fiorentino, Loredana, et al. "Increased tumor necrosis factor α–converting enzyme activity induces insulin resistance and hepatosteatosis in mice." *Hepatology* 51.1 (2010): 103-110.

Monroy, A., et al. "Impaired regulation of the TNF-α converting enzyme/tissue inhibitor of metalloproteinase 3 proteolytic system in skeletal muscle of obese type 2 diabetic patients: a new mechanism of insulin resistance in humans." *Diabetologia* 52.10 (2009): 2169-2181.

Cardellini, Marina, et al. "TIMP3 is reduced in atherosclerotic plaques from subjects with type 2 diabetes and increased by SirT1." *Diabetes* 58.10 (2009): 2396-2401.

Ewens, Kathryn Gogolin, et al. "Assessment of 115 candidate genes for diabetic nephropathy by transmission/disequilibrium test." *Diabetes* 54.11 (2005): 3305-3318.

1. Serino, Matteo, et al. "Mice heterozygous for tumor necrosis factor-α converting enzyme are protected from obesity-induced insulin resistance and diabetes." Diabetes 56.10 (2007): 2541-2546.
2. Gelling, Richard W., et al. "Deficiency of TNFα converting enzyme (TACE/ADAM17) causes a lean, hypermetabolic phenotype in mice." Endocrinology 149.12 (2008): 6053-6064.

Chang, Ying-Ju, et al. "The Rho-guanine nucleotide exchange factor PDZ-RhoGEF governs susceptibility to diet-induced obesity and type 2 diabetes." *Elife* 4 (2015).
