# Supplementary material for: Extracting proteins involved in disease progression using temporally connected networks
Source: BMC Syst Biol. 2018 Jul 25;12:78. doi: 10.1186/s12918-018-0600-z (PMC6060549; doi:10.1186/s12918-018-0600-z)
Supplement: Supplementary file 1 — Text S1. Mathematical representation of traversing a directed network. Validation of ranked lists from other temporal datasets. (ZIP 302 kb) [file 12918_2018_600_MOESM1_ESM.zip › Figure S1.pdf]

A

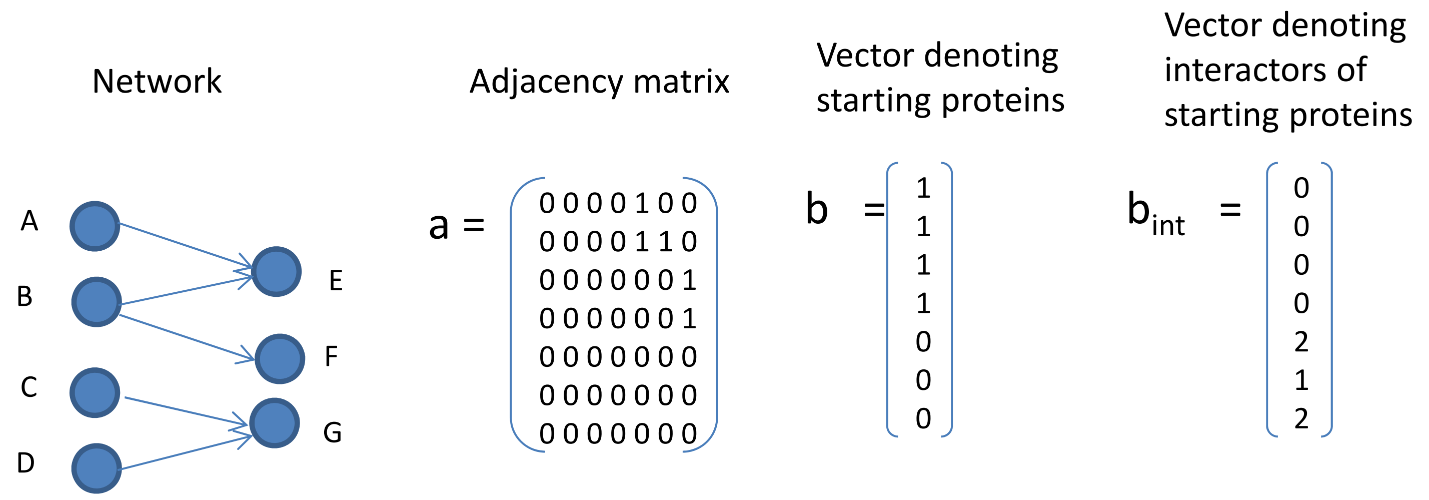

B

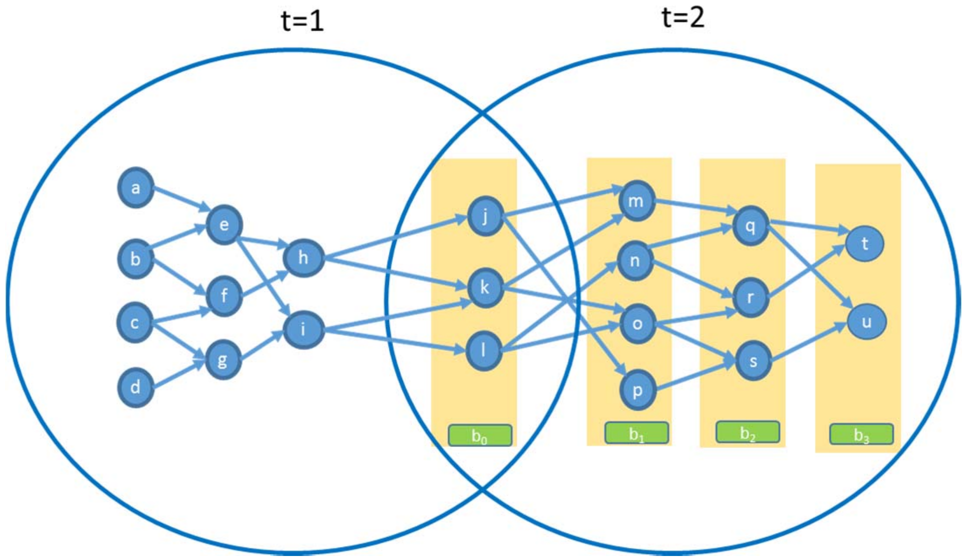

b_0, b_1, b_2 and b_3 are the vectors whose non-zero locations represents the nodes of each shaded regions i.e (j,k,l);(m,n,o,p);(q,r,s) and (t,u) respectively.Now,

$$B_f = b_0 + b_1 + b_2 + b_3$$

where,

$$b_1 = ({A_2}^T)b_0$$

$$b_2 = ({A_2}^T)b_1 = ({A_2}^T)({A_2}^T)b_0 = ({A_2}^T)^2 b_0$$

$$b_3 = ({A_2}^T)b_2 = ({A_2}^T)({A_2}^T)b_1 = ({A_2}^T)({A_2}^T)^2 b_0 = ({A_2}^T)^3 b_0$$

hence,

$$B_f = b_0 + ({A_2}^T)b_0 + ({A_2}^T)^2 b_0 + ({A_2}^T)^3 b_0$$

=proteins present in time point 2

$$= \begin{pmatrix} 0\\0\\0\\0\\0\\0\\0\\0\\0\\1\\1\\1\\0\\0\\0\\0\\0\\0\\0\\0\\0 \end{pmatrix} + \begin{pmatrix} 0\\0\\0\\0\\0\\0\\0\\0\\0\\0\\0\\0\\1\\2\\2\\1\\0\\0\\0\\0\\0 \end{pmatrix} + \begin{pmatrix} 0\\0\\0\\0\\0\\0\\0\\0\\0\\0\\0\\0\\0\\0\\0\\0\\3\\4\\3\\0\\0 \end{pmatrix} + \begin{pmatrix} 0\\0\\0\\0\\0\\0\\0\\0\\0\\0\\0\\0\\0\\0\\0\\0\\0\\0\\0\\3\\7 \end{pmatrix} = \begin{pmatrix} 0\\0\\0\\0\\0\\0\\0\\0\\0\\1\\1\\1\\1\\2\\2\\1\\3\\4\\3\\3\\7 \end{pmatrix}$$
