# Supplementary figures and images for: Extracting proteins involved in disease progression using temporally connected networks
Source: BMC Syst Biol. 2018 Jul 25;12:78. doi: 10.1186/s12918-018-0600-z (PMC6060549; doi:10.1186/s12918-018-0600-z)

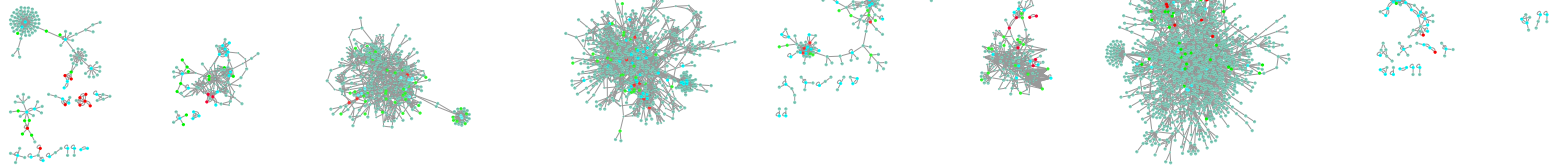

Supplement: Supplementary file 2 — Figure S2. High Resolution version of Fig. 1d. (PDF 644 kb) [file 12918_2018_600_MOESM2_ESM.pdf]

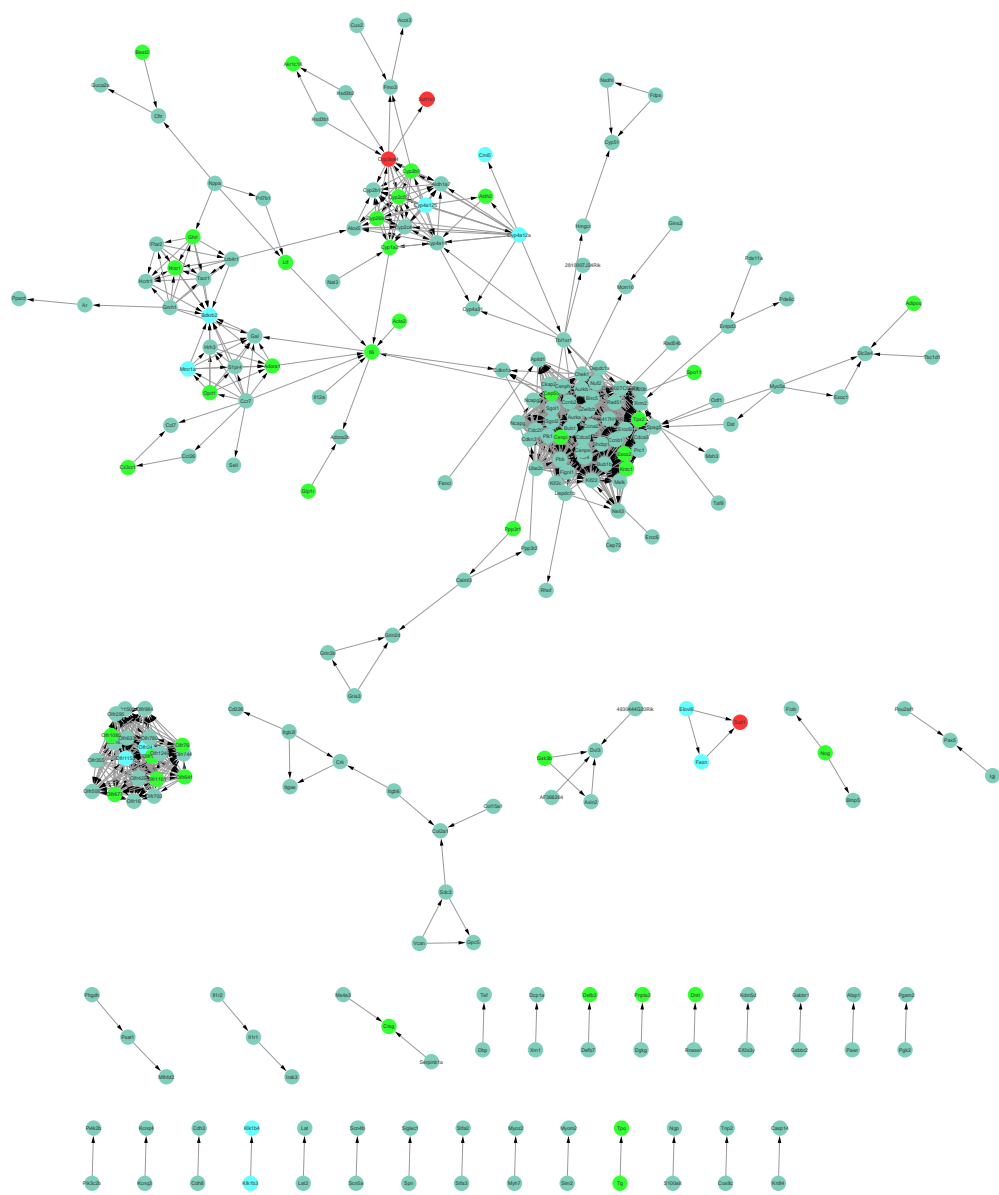

Supplement: Supplementary file 3 — Figure S3. High Resolution version of Fig. 2b. (PDF 108 kb) [file 12918_2018_600_MOESM3_ESM.pdf]

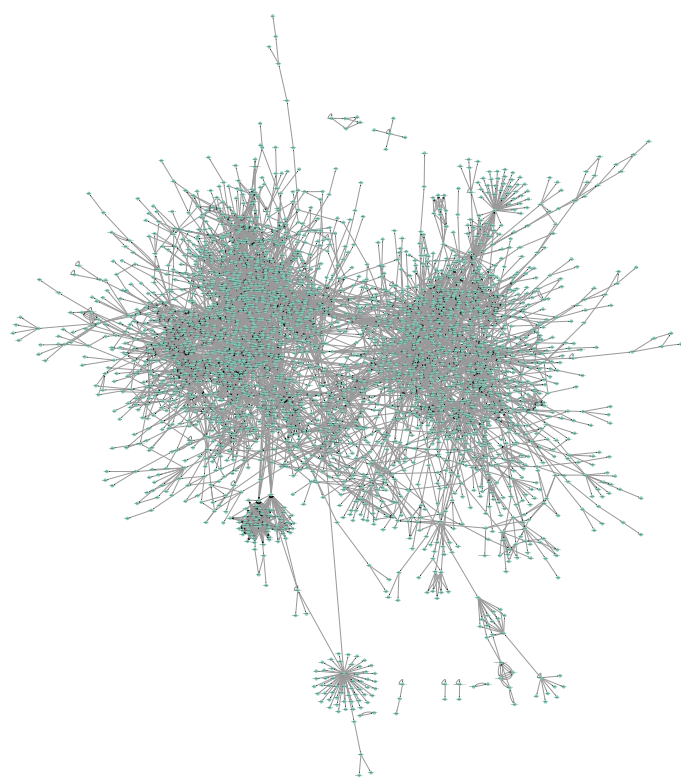

Supplement: Supplementary file 5 — Figure S4. High Resolution version of Fig. 2f. (PDF 817 kb) [file 12918_2018_600_MOESM5_ESM.pdf]

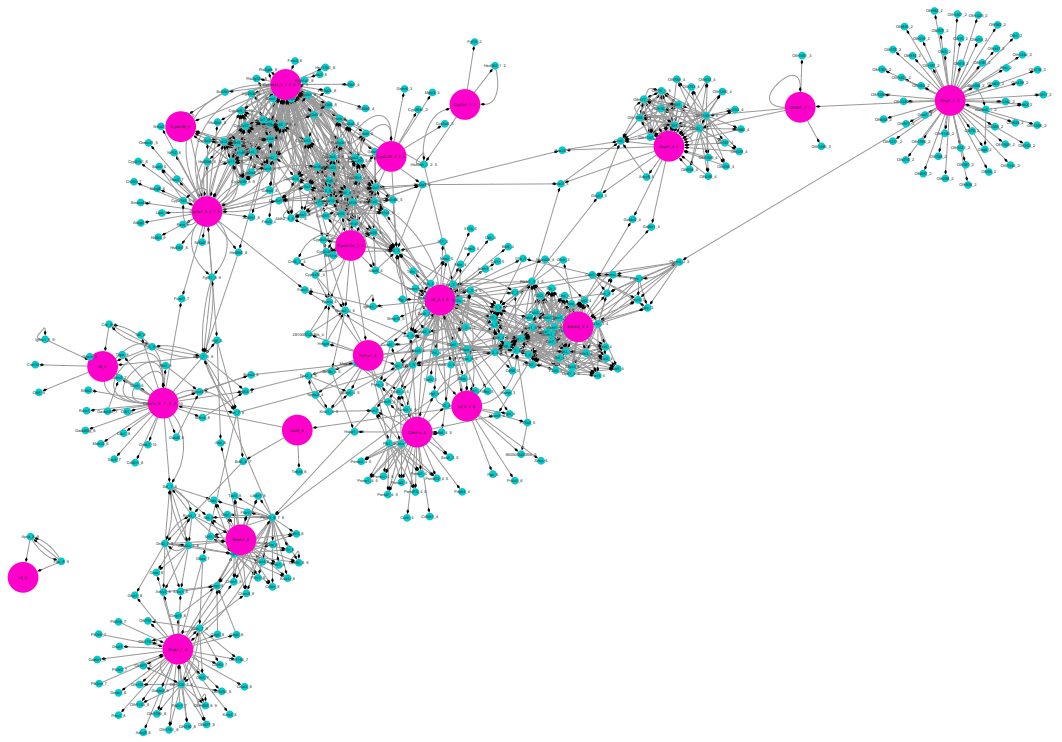

Supplement: Supplementary file 6 — Figure S5 High Resolution version of Fig. 2g left panel. (PDF 172 kb) [file 12918_2018_600_MOESM6_ESM.pdf]

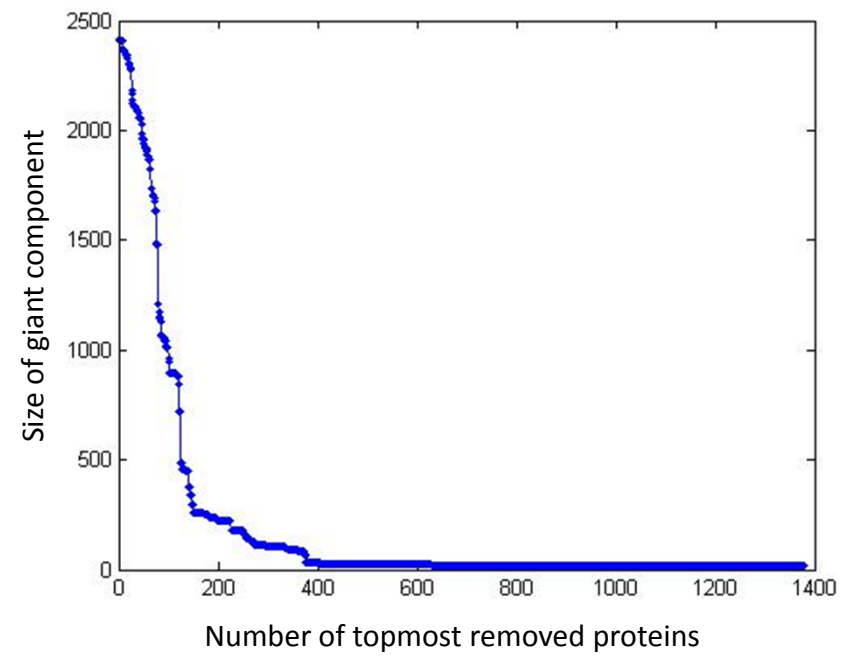

Supplement: Supplementary file 7 — Figure S6. Effect of removal of influencing proteins on size of giant component. Size of the giant component of single connected network on removal of topmost proteins from the ranked list sorted in decreasing order based on their influence obtained by applying ‘Collective Influence’ algorithm on single connected network. (PDF 2365 kb) [file 12918_2018_600_MOESM7_ESM.pdf]
