# Supplementary material for: Extracting proteins involved in disease progression using temporally connected networks
Source: BMC Syst Biol. 2018 Jul 25;12:78. doi: 10.1186/s12918-018-0600-z (PMC6060549; doi:10.1186/s12918-018-0600-z)
Supplement: Supplementary file 8 — Figure S7. Significance of overlap of topmost genes with positive gene set. The p-value of significance of overlap between topmost genes and positive gene set (fraction of times overlap from random permuted ranked list is greater than actual observed overlap) is plotted against topmost proteins used. (PDF 118 kb) [file 12918_2018_600_MOESM8_ESM.pdf]

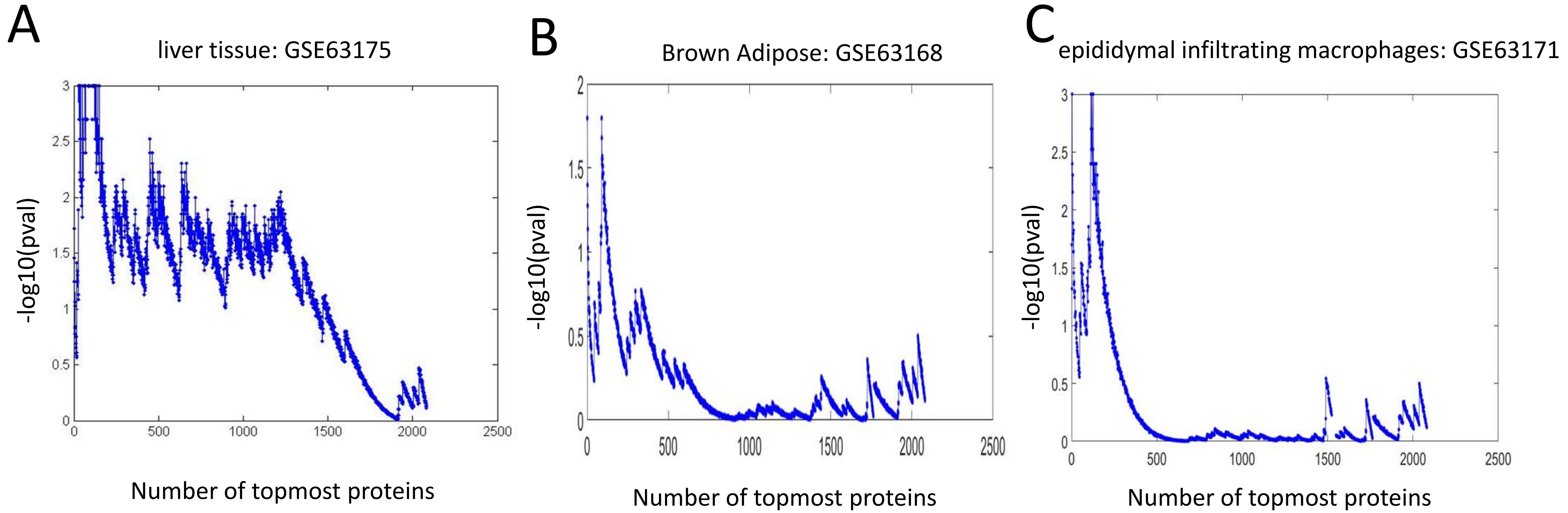
A
liver tissue: GSE63175
-log10(pval)
Number of topmost proteins
B
Brown Adipose: GSE63168
-log10(pval)
Number of topmost proteins
C
epididymal infiltrating macrophages: GSE63171
-log10(pval)
Number of topmost proteins
